# Development of Murine Hepatic NK Cells during Ontogeny: Comparison with Spleen NK Cells

**DOI:** 10.1155/2012/759765

**Published:** 2011-12-06

**Authors:** Xian Wu, Yongyan Chen, Haiming Wei, Rui Sun, Zhigang Tian

**Affiliations:** Institute of Immunology, Hefei National Laboratory for Physical Sciences at Microscale and School of Life Sciences, University of Science and Technology of China, Hefei 230027, China

## Abstract

The phenotype of developing liver NK cells (CD3^−^NK1.1^+^) was investigated during mouse ontogeny comparing with spleen NK cells. The highest percentage of hepatic CD27^−^CD11b^−^ NK cells occurred at the fetal stage. After birth, the percentage of CD27^−^CD11b^−^NK cells in both the liver and spleen gradually decreased to their lowest level at 6 weeks. More CD27^+^CD11b^−^NK cells were detected in the liver than that in spleen from week 1 to 6. Expression of NKG2A on liver NK cells was decreased but still much higher than that of spleen NK cells after 1 week. The NKG2D expression on liver NK cells increased to its highest level and was significantly higher than on spleen NK cells till 4 weeks. During mouse ontogeny, weaker expression of NKp46 and CD2 and stronger expression of CD69, CD11c, 2B4, and CD73 were observed on liver NK cells. Furthermore, neonatal liver NK cells express higher IFN-*γ* and perforin than adult .These results suggest that the maturation process of NK cells is unique in the livers, and liver microenvironments might play critical roles to keep NK cells in an immature status.

## 1. Introduction

NK cells are derived from haematopoietic stem cells (HSCs). The precursors of NK cells are generated in the bone marrow; they are committed to the NK cell lineage and develop into mature NK cells with full effector function and heterogeneous phenotypes [1, 2]. The definitive site(s) for NK cell development can only be inferred from where immature and mature NK cells have been detected. NK cell precursors (NKPs) are found in different organs, such as bone marrow, fetal thymus, lymph node (LN), liver, spleen, and peripheral blood, whereas immature NK (iNK) cells are found in the bone marrow, liver, and spleen [3]. It is unknown whether these developmental intermediates leave the bone marrow to complete their differentiation elsewhere, such as the liver and spleen.

 In liver, but not spleen, a unique subset of immature NK cells constitutively express tumour necrosis factor-related apoptosis-inducing ligand (TRAIL) and low levels of mature NK cell markers, such as the Ly49 receptors and CD11b [4–8]. A subset of NK-cells highly expressing CD11c have also been found specifically in the liver [9]. Adoptive transfer of either adult or neonatal mouse liver TRAIL^+^ NK cells results in the appearance of TRAIL^−^ NK cells with a mature phenotype, suggesting that these TRAIL^+^ NK cells in the liver were indeed a precursor subset [4]. Stromal cells in various organs send signals through cytokines, receptors, and transcription factors that influence the ultimate phenotypes and functions of NK cell precursors [2, 10–15], suggesting that there may be specific developmental pathways for intrahepatic NK cells. D. M. Andrews and M. J. Smyth have described differences in the accumulation of NK cell subsets in the liver, bone marrow, spleen, and lung between WT B6 mice and *Rag1^−/−^* mice during weeks 1–5 and at 8 weeks of age. Costaining of CD27 and CD11b were used to divide NK1.1^+^CD3^−^ NK cells into four subsets that were at different maturation stages [16]. The first appearance of mature CD27^−^CD11b^+^ NK cells in these organs, including bone marrow, spleen, and lung, occurs at 3 weeks of age, and maturation is complete by 8 weeks of age. Complete maturation of hepatic NK cells occurs at 2 weeks of age, with fewer CD27^−^CD11b^+^ NK cells accumulating in the adult mouse liver. These results demonstrate that the liver displays slower kinetics in the accumulation of terminally mature CD27^−^CD11b^+^ NK cells. Furthermore, in neonatal *Rag1^−/−^* mice, NK cells are absent in bone marrow and spleen, but a precursor NK cell subset is found in the liver, and normal NK cells without functional deficiencies can be detected in adult *Rag1^−/−^* mice. It was hypothesised that liver NK cells develop independently out of the bone marrow and that Rag-1 has a significant role in NK cell development [17, 18]. These results have helped us to understand the unique development pathway of liver NK cells; however, the details of phenotypes of developing liver NK cell subsets during mouse ontogeny have not been fully elucidated. 

In our study, NK cell development in liver was explored and compared with NK cell development in spleen during mouse ontogeny. We found an abundance of NKPs, but the development pathway did not occur concurrently in the liver and spleen. The CD27^−^CD11b^−^ NK cell precursors accumulated predominantly in the adult liver and not in the spleen. In the liver, more immature NK cells were present, which express a higher level of NKG2A and lower levels of Ly49 receptors. Additionally, different stimulatory receptors and adhesion molecules were expressed on NK cells in the liver and spleen during ontogeny. And the expression level of IFN-gamma and perforin were higher of neonatal liver NK cells comparing with 10-week-old liver NK cells. These results indicate that there might be a specific developmental pathway of NK cells in the liver and that the microenvironments play important roles in NK cell development and differentiation.

## 2. Results

### 2.1. Maturation of Liver NK Cells Is Different from That of Spleen NK Cells during Ontogeny

**Figure 1:**
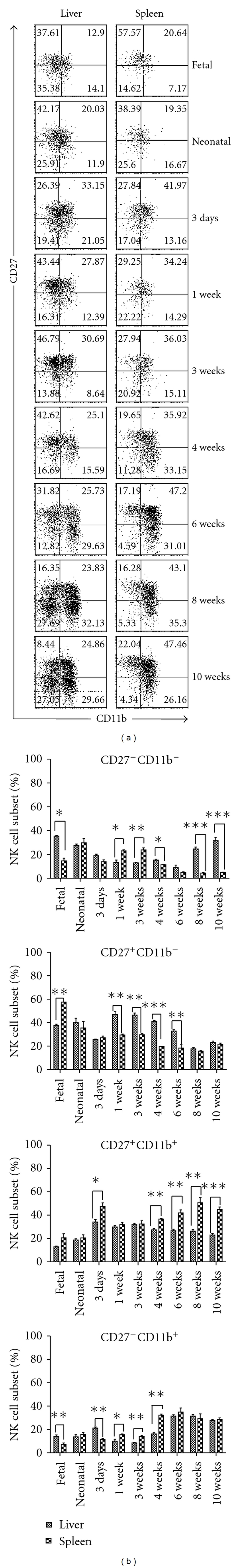
Different maturation stages of NK cells according to expression levels of CD27 and CD11b. Flow cytometry analysis was performed to analyse lymphocytes from the liver and spleen of B6 mice at the ages of E20, the neonatal stage, and at 3 days, 1 week, 3 weeks, 4 weeks, 6 weeks, 8 weeks, and 10 weeks, which were stained with the indicated antibodies. NK cells (CD3^−^NK1.1^+^) were gated to analyse the expression of CD27 and CD11b. Six to seven fetal mouse livers or spleens were put together to acquire enough cells to perform FACS analysis in one experiment, and three independent experiments were performed. In the other groups, there were three mice independently detected for one experiment, and three independent experiments were performed. (a) The percentages represent the net percentage (%) of cells in the appropriate quadrant. These are from a single experiment representative of three independent experiments. (b) The percentages of four NK cell subsets in the liver and spleen were calculated. Data are shown as the mean ± SEM from three mice in each group. ***P < 0.001, **P < 0.01, *P < 0.05.

Based on the expression of CD11b and CD27, NK cells (NK1.1^+^CD3^−^) can be divided into four subsets at different maturation stages [16, 19]. The gating strategy is shown in Figure 1 of Supplementary Material available at doi 10.1155/2012/759765. As the most immature subset, CD27^−^CD11b^−^ NK cells are the precursors of the other three NK cell subsets [16]. As shown in Figure 1, the highest percentage of CD27^−^CD11b^−^ NK cells in the liver occurred at embryonic day 20 (E20) (35.38 ± 0.64%). Comparatively, the percentage of CD27^−^CD11b^−^ NK cells was much lower in the spleen of E20 mice (14.62 ± 3.19%). However, the percentage of CD27^−^CD11b^−^ NK cells increased markedly to their highest level in the spleen of neonatal mice (29.73 ± 6.50%), which was similar to that found in the liver (Figure 1). As the mice growth, the percentage of CD27^−^CD11b^−^ NK cells in the spleen and liver decreased markedly, reaching a nadir at week 6 (4.91 ± 0.74% and 8.97 ± 3.51%, resp., Figure 1), but CD27^−^CD11b^−^ NK cells increased markedly in the liver to their highest levels once more at week 8 to 10 (24.64 ± 2.66% and 31.53 ± 5.13%, resp.). These results indicate that CD27^−^CD11b^−^ NK precursor cells reside predominantly in the adult liver and not in the spleen. 

Subsequently, in the liver of E20 mice, the percentage of immature CD27^+^CD11b^−^ NK cells was significantly lower than in the spleen (37.61 ± 1.51% versus 57.57 ± 0.007%, *P* = 0.0029, Figure 1). From week 1 to 6, there was higher percentage of CD27^+^CD11b^−^ NK cells in the liver compared with the spleen. At 8 weeks of age, no significant difference in the percentage of CD27^+^CD11b^−^ NK cells was found between the liver and spleen of adult mice. 

CD27^+^CD11b^+^ NK cells and CD27^−^CD11b^+^ NK cells are mature NK cells [5]. In the liver, the percentage of CD27^+^CD11b^+^ NK cells remained at a steady level, while in the spleen, this NK cell subset increased to a higher percentage during ontogeny. At 6 weeks of age, the percentage of CD27^+^CD11b^+^ NK cells in the spleen of the adult mice was 41.93 ± 4.58%, but the percentage of CD27^+^CD11b^+^ NK cells in the liver was only 26.73 ± 2.28% (Figure 1), which is similar to levels found in 8- to 10-week-old mice. In 3-day-old mice, the percentage of mature CD27^−^CD11b^+^ NK cells in the liver was higher than in the spleen (21.18 ± 1.67% versus 11.48 ± 1.51%, *P* = 0.0017, Figure 1), but from weeks 1 to 4, the percentages in the liver and spleen were reversed. In the 6-week-old adult mice, the percentages of CD27^−^CD11b^+^ NK cells in the liver and spleen were similar (31.48 ± 1.86 and 34.85 ± 6.17, resp., *P* = 0.4161, Figure 1). 

These results indicate that the maturation process is different in liver and spleen. In each organ of neonatal mice, there was a high percentage of NK cell precursors, but the liver NK cell development did not occur in parallel to spleen NK cells. In the liver, there were more immature NK cells. 

### 2.2. Developing NK Cells Expresses Different Inhibitory and Stimulatory Receptors in the Livers When Compared with Spleen during Ontogeny

**Figure 2:**
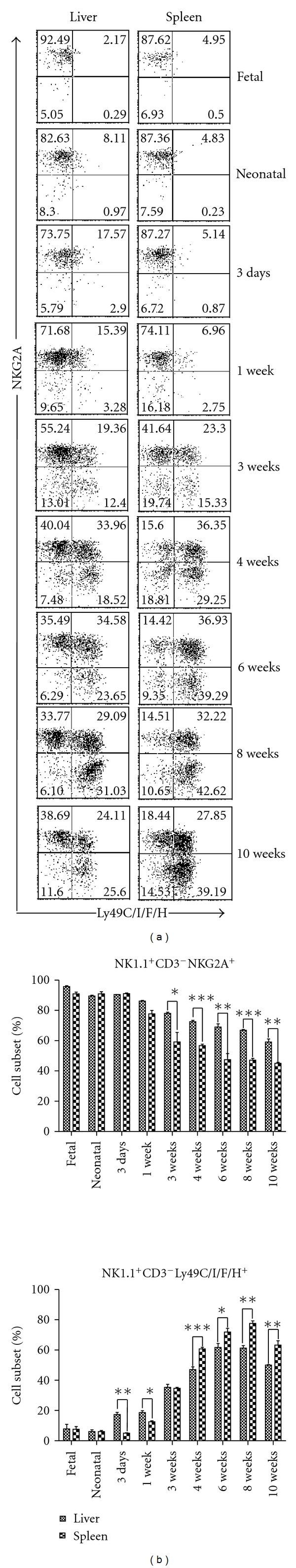
Different expression patterns of the NKG2A/Ly49 family of receptors on NK cells during mouse ontogeny. Flow cytometry was performed to analyse the lymphocytes stained with indicated antibodies from liver and spleen of B6 mice at E20, the neonatal stage, and at 3 days, 1 week, 3 weeks, 4 weeks, 6 weeks, 8 weeks, and 10 weeks. NK cells (CD3^−^NK1.1^+^) were gated to analyse the expression of NKG2A and Ly49. In each independent experiment, six to seven fetal mouse livers/spleens were put together to acquire enough cells to perform FACS analysis, and independent experiments were repeated for three times. (a) The percentages represent the net percentage (%) of cells in the appropriate quadrant. These are from a single experiment representative of three independent experiments. (b) The percentages of the NKG2A^+^ NK cell subset (%) and the Ly49^+^ NK cell subset (%) were calculated from the total number of NK cells in the liver and spleen. Data are shown as the mean ± SEM from three mice in each group. ***P < 0.001, **P < 0.01, *P < 0.05 compared with the corresponding group.

As shown in Figures 2(a) and 2(b), at the earliest stage of development, almost all NK cells were NKG2A-positive in the liver and spleen (95.71 ± 1.07% and 90.98 ± 1.95%, resp., Figure 2). With further development, the percentage of NKG2A^+^ NK cells gradually decreased, but the decrease in liver was delayed compared with spleen. In 10-week-old adult mice, 59.16 ± 3.36% of NK cells were NKG2A^+^ in liver, compared to 45.05 ± 1.11% in spleen (Figure 2). NK cells gradually acquired expression of Ly49 receptors, which are markers delineating the maturation stages of NK cells [3]. In the livers from fetal and neonatal mice, only 7.81 ± 5.31% and 6.04 ± 1.98% of NK cells, respectively, were Ly49C/I/F/H^+^ (Figure 2). Of note, in 3-day-old mice, the percentage of Ly49C/I/F/H^+^ NK cells in the liver rapidly increased to 17.36 ± 2.50% (Figure 2), which was significantly higher than in spleen (5.01 ± 0.61%, Figure 2). However, at 1 week the expression of Ly49 C/I/F/H on spleen NK cells rapidly increased, and after 4 weeks, the expression level in the liver was markedly lower than in the spleen. These results further indicate that the development of NK cells did not occur concurrently. In the liver, NK cells had phenotypes characteristic of immature subsets. 

**Figure 3:**
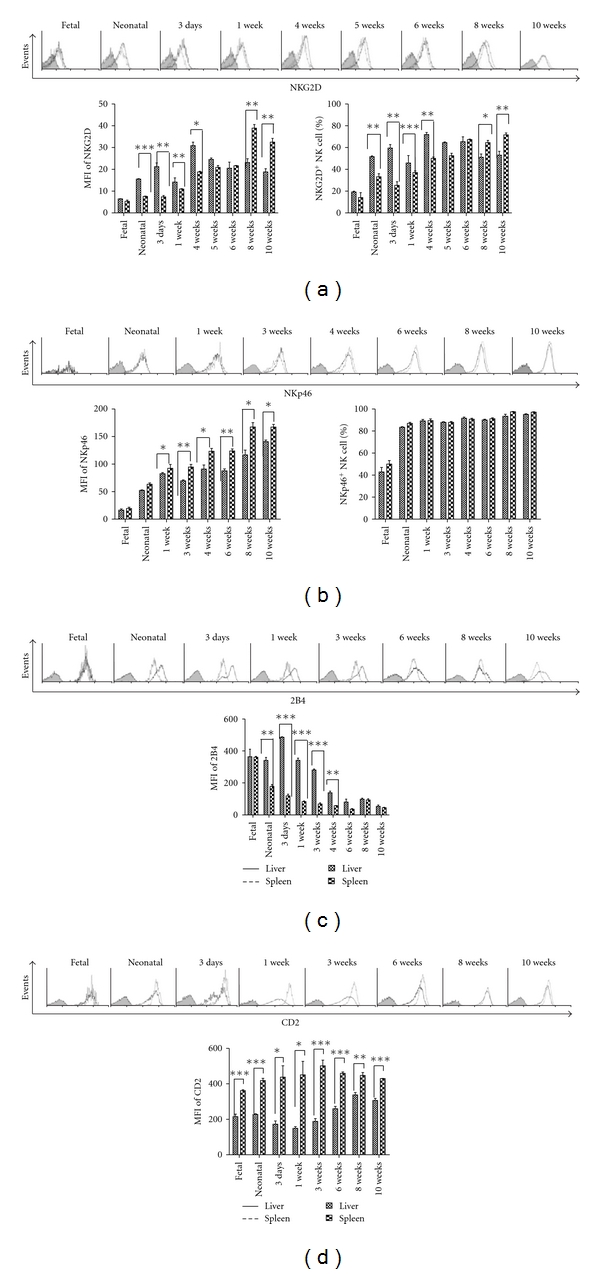
Stimulatory receptors expressed on NK cells during mouse ontogeny. At the indicated time points, mononuclear cells were isolated from liver and spleen and analysed by flow cytometry for the expression of stimulatory receptors. NK cells (NK1.1^+^CD3^−^) were gated, and each marker was analysed by histogram. Unstained controls are shown in grey. The expression of NKG2D (a), NKp46 (b), 2B4 (c), and CD2 (d) on liver NK cells is shown and compared with spleen NK cells during ontogeny. In each independent experiment, six to seven fetal mouse livers or spleens were put together to acquire enough cells to perform FACS analysis in one experiment, and three independent experiments were performed. In the other groups, there were three mice independently detected for one experiment, and three independent experiments were performed. The mean fluorescence intensity (MFI) and positive percentage of each marker are shown as mean ± SEM from three mice in each group. These are from a single experiment representative of three independent experiments. ***P < 0.001, **P < 0.01, *P < 0.05 compared with the corresponding group.

To further investigate the functions of NK cells at different developmental stages in the liver and spleen, a series of stimulatory receptors was detected by flow cytometry. From the mean fluorescence intensity and percentage in both liver and spleen of fetal and neonatal mice, the expression of NKG2D on NK cells was very low (Figure 3(a)). At later developmental stages, the expression of NKG2D was upregulated. In liver, the expression of NKG2D increased to its highest level at 4 weeks and was significantly higher than in spleen, while in spleen, NKG2D increased to its highest level at 8 weeks. This result indicates that earlier expression of the stimulatory NKG2D receptor in hepatic NK cells might be associated with their specific function in the liver. At 8 to 10 weeks, the expression level of NKG2D on liver NK cells (51.14 ± 5.18% and 53.01 ± 6.2%, resp.) was significantly lower than in spleen (64.56 ± 3.58% and 71.68 ± 3.10%, resp., Figure 3(a)). As shown in Figure 3(b), there was no significant difference in the percentage of NKp46-expressing NK cells between liver and spleen. From newborn to adult mice, NKp46 was expressed on almost all NK cells in the liver and spleen, but the fluorescence intensity of NKp46 expression was much weaker in the liver compared with the spleen.

 In addition, we found that all NK cells in both the liver and spleen expressed 2B4 and CD2; however, the fluorescence intensity differed between the two tissues (Figures 3(c) and 3(d)). In the earlier stage of ontogeny, from the fetal stage to 3 weeks after birth, the expression level of 2B4 on NK cells in liver was consistent with a high MFI, while 2B4 expression on NK cells in spleen decreased markedly after the neonatal stage (Figure 3(c)). At 6 weeks of age, the expression of 2B4 on NK cells in adult mice decreased to a nadir in both liver and spleen. During ontogeny, the expression of CD2 on NK cells in liver and spleen was relatively stable, and the expression of CD2 on NK cells in the liver was significantly lower than in the spleen (Figure 3(d)). Different inhibitory and stimulatory receptors were expressed on NK cells in the liver compared with the spleen during ontogeny, which may be related to the specific functions of NK cells in different organs.

### 2.3. Higher Expression of Function-Related Molecules on Liver NK Cells Compared with Spleen NK Cells during Ontogeny

**Figure 4:**
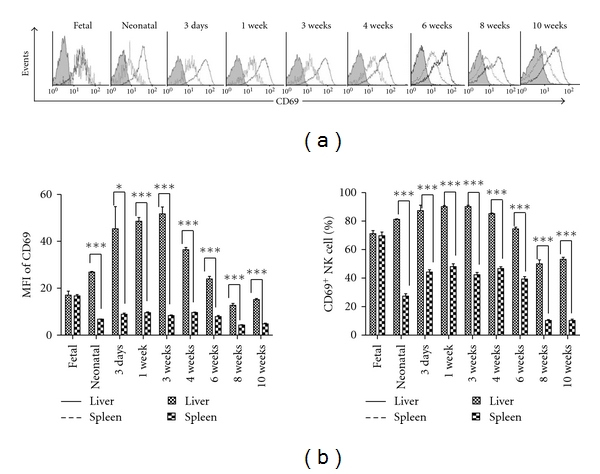
The expression level of CD69 on liver NK cells is higher than on spleen NK. Flow cytometry was performed to analyse lymphocytes stained with the indicated antibodies from liver and spleen of B6 mice at E20, the neonatal stage, and at 3 days, 1 week, 3 weeks, 4 weeks, 6 weeks, 8 weeks, and 10 weeks. NK cells (CD3^−^NK1.1^+^) were gated to analyse the expression of CD69. In each independent experiment, six to seven fetal mouse livers or spleens were put together to acquire enough cells to perform FACS analysis in one experiment, and three independent experiments were performed. In the other groups, there were three mice independently detected for one experiment, and three independent experiments were performed. (a) Each percentage represents the net percentage (%) of cells in the appropriate quadrant. These results are from a single experiment representative of three independent experiments. (b) The percentages of the CD69^+^ NK cell subset (%) were calculated for the total number of NK cells in the liver and spleen. Data are shown as mean ± SEM from three mice in each group. ***P < 0.001, **P < 0.01, *P < 0.05 compared with the corresponding group.

In the foetus, liver and spleen NK cells exhibited elevated expression of CD69 (71.18 ± 3.05% and 69.75 ± 3.68%, resp., Figure 4). At later stages of development, the expression of CD69 on NK cells in the liver was upregulated to reach a maximum at 3 weeks, while in the spleen, the expression was downregulated after the neonatal stage. Although the percentage of CD69-expressing NK cells in the liver decreased to 50.07 ± 4.65% at 8 weeks of age, it remained significantly higher than in the spleen (10.33 ±1.14%); similar percentages were found in 10-week-old mice (53.23 ± 2.42% and 10.29 ± 1.62%, resp., *P* < 0.0001, Figure 4). The results obtained with dynamic MFI further confirmed higher expression of CD69 on liver NK cells during mouse ontogeny (Figure 4(b)).

**Figure 5:**
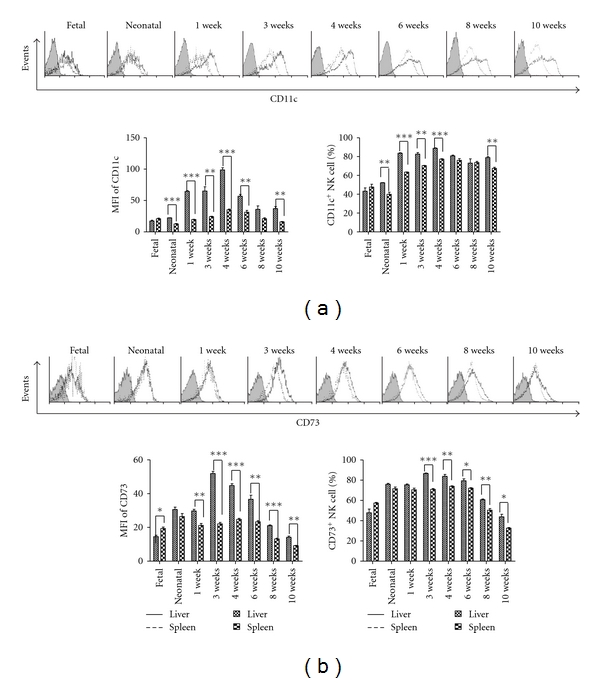
Adhesion molecules expressed on NK cells during mouse ontogeny. At the indicated time points, mononuclear cells were isolated from the liver and spleen and analysed by flow cytometry for the expression of adhesion molecules. NK cells (NK1.1^+^CD3^−^) were gated, and each marker was analysed by histogram. In each independent experiment, six to seven fetal mouse livers or spleens were put together to acquire enough cells to perform FACS analysis in one experiment, and three independent experiments were performed. In the other groups, there were three mice independently detected for one experiment, and three independent experiments were performed. The expression of CD11c (a) and CD73 (b) on liver NK cells is shown and compared with spleen NK cells during ontogeny. The mean fluorescence intensity (MFI) and percentage of each marker is shown as the mean ± SEM from three mice in each group. These are from a single experiment representative of three independent experiments. ***P < 0.001, **P < 0.01, *P < 0.05 compared with the corresponding group.

 Additionally, the differential expression pattern of adhesion molecules distinguished liver NK cells from spleen NK cells. CD11c was expressed more highly on liver NK cells than on spleen NK cells during mouse ontogeny, except in fetal mice (Figure 5(a)). The highest level of CD11c on liver NK cells was observed at 4 weeks, but it decreased in adult mice (Figure 5(a)). Similarly, CD73 was expressed more highly on liver NK cells, reaching its highest level at 3 weeks (Figure 5(b)). 

**Figure 6:**
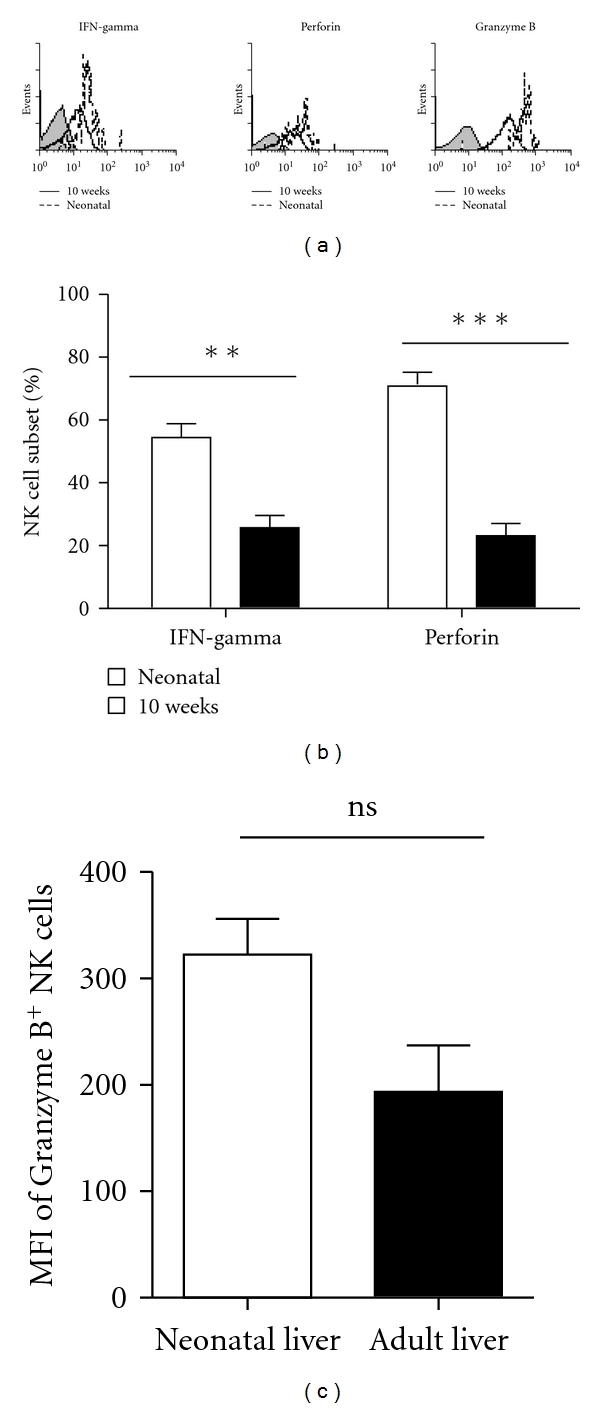
Neonatal liver NK cells produced more IFN-gamma, perforin, and Granzyme B than 10-week-old liver NK cells after stimulated with Poly I: C in vitro. In each independent experiment, six to seven neonatal mouse livers/spleens were put together to acquire enough cells to perform FACS analysis. NK cells (NK1.1^+^CD3^−^) were gated, and then each marker was analyzed by histogram. Unstained controls are the grey. (a) Each percentage represents the net percentage (%) of cells in the appropriate quadrant. (b) The percentages of the IFN-gamma^+^ NK cell subset (%) were calculated for the total number of NK cells in the liver and spleen. Data are shown as mean ± SEM three mice in each group. ***P < 0.001, **P < 0.01, *P < 0.05 compared with the corresponding group.

 To further explore the functions of hepatic NK cells at different developmental stages, hepatic MNCs were stimulated with Poly I : C in vitro, and then the expressions of IFN-gamma, perforin, and Granzyme B of NK cells were tested. After stimulated with Poly I : C in vitro, there were more IFN-gamma and perforin expressed by the neonatal liver NK cells than 10-week old liver NK cells (*P* = 0.0082 and *P* = 0.0009) (Figure 6). However, there were no significant differences of the expression of GranzymeB between neonatal liver NK cells and 10-week-old liver NK cells (Figure 6). It suggested that the function of neonatal liver NK cells may stranger than adult liver NK cells.

## 3. Discussion

The phenotypes and functions of NK cells change with age and location [12]. There is a unique intrahepatic NK cell subset with the immature phenotype of NKG2A^+^Ly49s^−^DX5^−^TRAIL^+^ [4–6, 8, 17, 18]. A large body of evidence has supported the existence of a specific development pathway of NK cells in liver [4, 17, 18]. In our study, we described the development of NK cells in liver compared with spleen during mouse ontogeny.

There is a significantly higher percentage of the CD27^−^CD11b^−^ NK cell subset in liver than in spleen in adult wild-type C57BL/6 mice [18–20], which we confirmed in our study (Figure 1). Furthermore, our study is the first to describe the presence of a high percentage of the CD27^−^CD11b^−^ NK cell subset in fetal mouse liver and to demonstrate that the percentage of this NK cell subset was persistently elevated in the adult liver during ontogeny (Figure 1). Because the fetal liver is the major haematopoietic organ during embryogenesis, we speculate that the CD27^−^CD11b^−^ NK cell subset originated from the fetal liver. The NK cell population is absent in bone marrow and spleen from neonates of *Rag1^−/−^* mice but accumulates in bone marrow and spleen of adult mice. Additionally, an overrepresentation of CD27^−^CD11b^−^ NK cells, which are considered a precursor NK cell subset found normally in the liver, is observed in the bone marrow of *Rag1^−/−^* mice. This suggests that liver NK cell precursors might seed into other organs to compensate for the absence of bone marrow-derived NK cells [17]. The predominant NK cell subsets in the spleen were of the mature phenotypes, CD27^+^CD11b^+^ and CD27^−^CD11b^+^(Figure 1), which differed from liver NK cells during mouse ontogeny. The CD27^−^CD11b^−^ NK cells were the most immature NK cells which can develop into the other three subset NK cells. As reported, NK cells can develop along DN (CD27^−^CD11b^−^) → CD27^+^CD11b^−^ → DP (CD27^+^CD11b^+^) → CD27^−^CD11b^+^model [16]. However, studies of physiological functions of these four NK subsets were limited. It has been reported that CD27^+^CD11b^+^ exhibited stronger cytotoxicity and produced more IFN-*γ* than CD27^−^CD11b^+^ NK cells in response to cytokine stimulation [19]. In the liver, there may be a unique developmental pathway distinct from the spleen such that NK cell subsets at different maturation stages distribute specifically to liver and spleen during mouse ontogeny. 

The inhibitory NKG2A and Ly49 receptors can be considered markers of the NK cell maturation stage. In fetal and neonatal spleen, almost all NK cells express NKG2A, and the percentage of NKG2A^+^ NK cells decreases during mouse ontogeny [20–22], however, NK cells do not express Ly49C/I/F/H in early development and gradually acquire these receptors [21, 23, 24]. The same kinetics of the expression levels of NKG2A and Ly49 receptors were observed on spleen NK cells during mouse ontogeny in our study (Figure 2). Furthermore, for the first time, we demonstrated that almost no Ly49C/I/F/H was expressed on liver NK cells in fetal and neonatal mice (Figure 2) and that the decrease of NKG2A^+^ NK cells and the increase of Ly49C/I/F/H^+^ NK cells were much slower in the liver than in the spleen during mouse ontogeny. This resulted in a higher percentage of NKG2A^+^ NK cells and a lower percentage of Ly49^+^ NK cells in liver compared to spleen (Figure 2(b)). These results further indicate that the development of NK cells does not occur concurrently in the liver and spleen. In the liver, NK cells displayed predominantly immature phenotypes. 

 During mouse ontogeny, we observed that in the livers of 3-day-old mice, the percentage of CD27^−^CD11b^−^ NK cells was comparatively lower than in fetal and neonatal mice, while the percentage of CD27^−^CD11b^+^ NK cells was higher in the 3-day-old mice (Figure 1). Accordingly, in the liver of 3-day-old mice, NK cells had acquired the expression of Ly49 receptors, occurring earlier than in spleen NK cells (Figure 2). These results indicate that NK cells in the liver undergo a rapid progression of development and differentiation after birth. In view of special double blood supplies from the hepatic artery and the portal vein, the liver is continuously exposed to large amounts of intestinal antigens after birth [25–27]. The development of liver NK cells during early mouse ontogeny might relate to the special physiological functions of the liver. We found that the expression of NKG2D rapidly increased to its highest level at 4 weeks and that the expression of CD69 was upregulated to its highest level at 3 weeks, indicating an activated phenotype of liver NK cells. However, intrahepatic NK cells expressed lower levels of NKp46 and CD2 if compared with spleen NK cells during mouse ontogeny (Figures 3 and 4). The liver with specific blood supply from intestines continuously encounters bacterial products and food-derived antigens. The liver must eliminate the blood toxic waste products and endotoxins or other bacterial degradation products from the gut, without eliciting an immune response in the normal condition, so the liver acts as a complex immune organ, functioning as a site of effective immune responses or of tolerance appropriately [28, 29]. The constitutive presence of non-self and microbial molecules may result in the activated statement of hepatic NK cells, which is related to the liver tolerance. It was evidenced that the liver's resident immune cells exist in a state of active tolerance and this state of tolerance may be reversed by the right combinational administration with immunostimulants. Furthermore, it has been speculated that the high content of organ-specific NK cells might be associated with liver immune tolerance. In humans, it has been proposed that the unique properties of the transferred hepatic NK cells from a donor may enable them to play a role in regulating the immunological response of the recipient against the graft and therefore contribute to liver tolerogenicity after liver transplantation [30].

The liver is a lymphoid organ with a predominantly innate immune system [31, 32]. NK cells are abundant in the normal liver, accounting for approximately one-third of intrahepatic lymphocytes, which differs from other lymphoid organs and peripheral blood [25, 33, 34]. NK cells sequentially express different integrins over the course of development and maturation [5] and alter their expression of integrins and chemotactic receptors for their redistribution from the bone marrow and lymph nodes to blood, spleen, liver, and lung [35]. In our study, we found a constantly elevated level of CD11c and CD73 expression on liver NK cells compared to spleen NK cells during mouse ontogeny (Figures 5(a) and 5(b)). These adhesion molecules may play a role in intrahepatic NK cell adherence and retention in the liver.

In this study, the development of intrahepatic NK cells was described and compared to spleen NK cells during mouse ontogeny. Our results indicate that in the liver, there might be a specific developmental pathway of NK cells and that the microenvironments play important roles in NK cell development and differentiation. Further research on the mechanisms of differentiation and activation, chemoattraction, adhesion, and functions of hepatic NK cells is warranted.

## 4. Materials and Methods

### 4.1. Animals

C57BL/6 mice were obtained from the Shanghai Experimental Animal Center (Shanghai, China) and maintained under specific pathogen-free and controlled conditions (22°C, 55% humidity, and 12-hour day/night rhythm). The animal experiments were performed in compliance with the guidelines outlined in the Guide for the Care and Use of Laboratory Animals. All procedures were in compliance with the regulations of animal care of University of Science and Technology of China. The accreditation number of the laboratory is SYXK (Anhui) 2005-004 from Anhui Science and Technology Department. All the surgery was performed under sodium pentobarbital anesthesia, and all efforts were made to minimize suffering. To obtain timed pregnant mice, mice were mated for 15 hours, and then at E20 (plug date = day 0) the foetuses were acquired. Neonatal mice were 24 hours old. 

The protocols regarding the use and care of animals in the research as described by the paper had been reviewed and approved by *the Intuitional Animal Care and Use Committee of University of Science and Technology of China* (the date of approval: March 15, 2009, the reference number of approval: USTCAU200900005). 

### 4.2. Isolation of Liver Mononuclear Cells

Liver mononuclear cells (MNCs) were isolated essentially as described previously [36]. Briefly, mouse liver was removed and pressed through a 200-gauge stainless steel mesh. The liver cell suspension was collected and then centrifuged at 50 g for 1 min. Supernatants containing MNCs were collected and washed in phosphate-buffered saline (PBS). The cells were resuspended in 40% Percoll (GE Healthcare) and then gently overlaid on 70% Percoll and centrifuged at 1260 g for 30 minutes at room temperature. The interface cells between the Percoll solutions were aspirated and washed twice in PBS. Six to seven fetal mouse livers were harvested together, and the liver MNCs were isolated as described above.

### 4.3. Isolation of Splenocytes

Mouse spleen was removed and pressed through a 200-gauge stainless steel mesh. The cell suspension was collected and centrifuged at 890 g for 10 min, and then the cells were subjected to red blood cell lysis before incubation in PBS. Six to seven fetal mouse spleens were harvested together for splenocyte isolation.

### 4.4. Flow Cytometry Analysis

For the intracellular cytokine assay, MNCs were cultured in the presence of 6 uM monensin (Sigma Chemical Co., St. Louis, MO) for 4 h in humidified 5% CO_2_ at 37°C. After blocking with anti-FcR, cells were subsequently stained with a saturating amount of the indicated fluorescence-labelled antibodies for 30 min at 4°C in darkness for the surface antigens. Subsequently, cells were fixed and permeabilized using 100 uL of cytofix and cytoperm solution (eBioscience, San Diego, Calif, USA), respectively, and then stained with the indicated fluorescence-labelled antibodies for 1 hour at 4°C in darkness for the intracellular antigens. Stained cells were washed twice in PBS and then acquired with an LSRII (Becton Dickinson) and analysed with WinMDI 2.9 software. 

### 4.5. Reagents

The fluorescence-labelled antibodies used in this study included FITC-anti-CD69 (clone H12F3), FITC-anti-CD11c (clone HL3), FITC-anti-CD11b (clone M1/70), FITC-anti-AHIgG1, FITC-anti-RatIgG2b, PE-anti-CD27 (clone LG.3A10), PE-anti-NKG2D (clone CX5), PE-anti-CD244 (clone 2B4), PE-anti-CD2 (clone RM2-5), PE-anti-AHIgG1, PE-anti-RatIgG2a, PE-anti-RatIgG1, PE-anti-MsIgG2b, PE-anti-RatIgG2b, PE-anti-IFN-gamma (clone XMG1.2), PerCP-Cy5.5-anti-NK1.1, PerCP-Cy5.5-anti-MsIgG2a, APC-anti-CD3e (clone 1452C11), APC-anti-AHIgG1, APC-anti-IgM, Alexa647-anti-RatIgG2a, APC-Cy7-anti-CD3e (clone 145-2C11), APC-Cy7-anti-AHIgG1 (BD Pharmingen, San Diego, Calif, USA), PE-anti-NKG2A (clone 16a11), FITC-anti-Ly49C/I/F/H (clone 14B11), PE-anti-CD73 (clone TY/11.8), PE-anti-perforin (clone eBioOMAK-D), PE-anti-Granzyme B (clone 16G6) and Alexa647-anti-NKp46 (eBioscience, San Diego, Calif, USA). RBC lysis buffer was purchased from Biolegend (San Diego, Calif, USA). 

### 4.6. Statistical Analysis

The results were analysed by Student's *t*-test, performed with GraphPad Prism v5.00 software. All data are shown as the mean ± standard error of the mean (SEM). *P* < 0.05 was considered statistically significant. 

## Supplementary Material

NK cells express a combination of antigens on their cell surface that can be recognized by specific antibodies. Multiple NK cell subpopulations can be determined by multiplex antibody-staining. This supplementary figure describes the gating strategies to analyze the data of NK cell subpopulations detected by a multi-parameter flow cytometer.

## Abbreviations

HSC: Haematopoietic stem cell
NK cell: Nature killer cell
NKP: Nature killer cell precursor
LN: Lymph node
iNK cell: Immature NK cell
TRAIL: Tumour necrosis factor-related apoptosis-inducing ligand
Rag: Recombination-activating gene
MFI: Mean fluorescence intensity
PBS: Phosphate-buffered saline
MNCs: Mononuclear cells
RBC: Red blood cells.
