# Supplementary material for: Development of Murine Hepatic NK Cells during Ontogeny: Comparison with Spleen NK Cells
Source: Clin Dev Immunol. 2011 Dec 6;2012:759765. doi: 10.1155/2012/759765 (PMC3235455; doi:10.1155/2012/759765)

**Supplementary Figure.1**

The gating strategies to detect mouse NK cell subsets. Lymphocytes are gated on R1 between FSC and FSC. The lymphocytes population is then shown with NK1.1 and CD3, NK cells are gated as NK1.1^+^ and CD3^−^ (R3). The first panel shows lymphocytes on which only ArH IgG1 PE and Rat IgG2b FITC isotypes were stained, while in the second panel CD11b is stained. The third panel shows lymphocytes on which only CD27 is stained. This allows the quadrant gates to be set appropriately on CD11b^+^ and CD27^+^.The final panel in part B shows the four major subsets (CD27^−^CD11b^−^ **a**, CD27^+^/CD11b^−^ **b**; CD27^+^/CD11b^+^ **c**; CD27^−^/CD11b^+^ **d**). The gating strategy was the same for all samples.


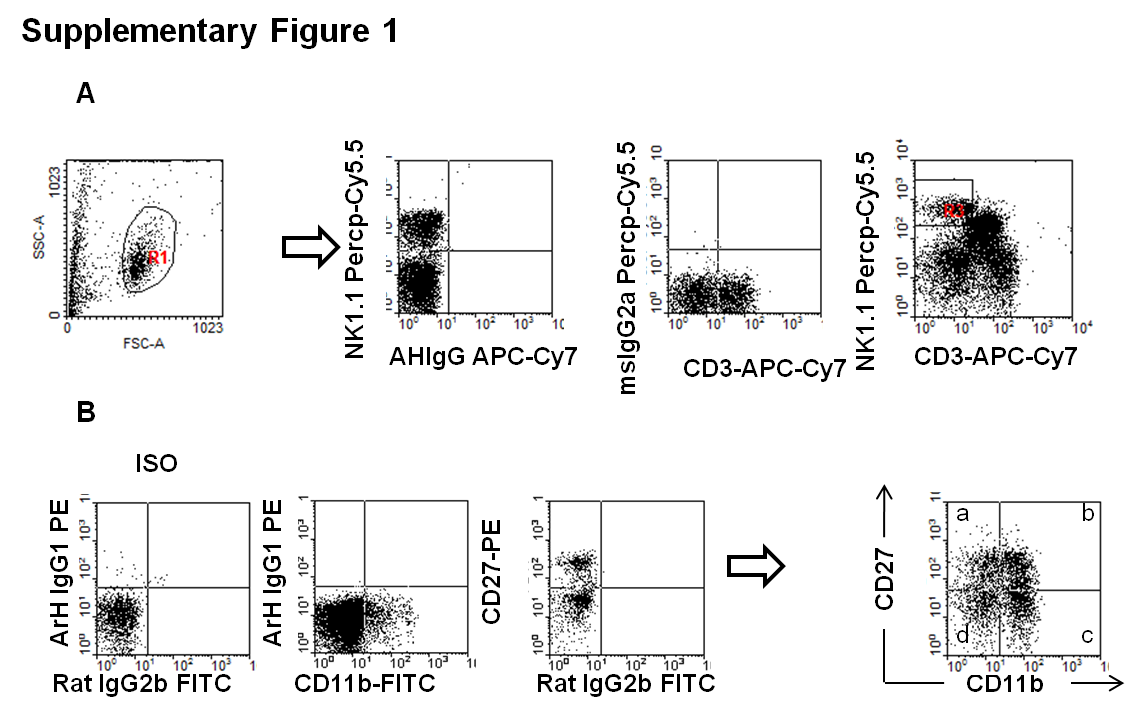

Supplement: Supplementary file 1 — NK cells express a combination of antigens on their cell surface that can be recognized by specific antibodies. Multiple NK cell subpopulations can be determined by multiplex antibody-staining. This supplementary figure describes the gating strategies to analyze the data of NK cell subpopulations detected by a multi-parameter flow cytometer. [file 759765.f1.docx]
